# Integrated Fossil and Molecular Data Reveal the Biogeographic Diversification of the Eastern Asian-Eastern North American Disjunct Hickory Genus (*Carya* Nutt.)

**DOI:** 10.1371/journal.pone.0070449

**Published:** 2013-07-16

**Authors:** Jing-Bo Zhang, Rui-Qi Li, Xiao-Guo Xiang, Steven R. Manchester, Li Lin, Wei Wang, Jun Wen, Zhi-Duan Chen

**Affiliations:** 1 State Key Laboratory of Systematic and Evolutionary Botany, Institute of Botany, Chinese Academy of Sciences, Beijing, China; 2 Florida Museum of Natural History, University of Florida, Gainesville, Florida, United States of America; 3 Department of Botany, National Museum of Natural History, Smithsonian Institution, Washington, D.C., United States of America; Chinese Academy of Sciences, China

## Abstract

The hickory genus (*Carya*) contains ca. 17 species distributed in subtropical and tropical regions of eastern Asia and subtropical to temperate regions of eastern North America. Previously, the phylogenetic relationships between eastern Asian and eastern North American species of *Carya* were not fully confirmed even with an extensive sampling, biogeographic and diversification patterns had thus never been investigated in a phylogenetic context. We sampled 17 species of *Carya* and 15 species representing all other genera of the Juglandaceae as outgroups, with eight nuclear and plastid loci to reconstruct the phylogeny of *Carya*. The phylogenetic positions of seven extinct genera of the Juglandaceae were inferred using morphological characters and the molecular phylogeny as a backbone constraint. Divergence times within *Carya* were estimated with relaxed Bayesian dating. Biogeographic analyses were performed in DIVA and LAGRANGE. Diversification rates were inferred by LASER and APE packages. Our results support two major clades within *Carya*, corresponding to the lineages of eastern Asia and eastern North America. The split between the two disjunct clades is estimated to be 21.58 (95% HPD 11.07-35.51) Ma. Genus-level DIVA and LAGRANGE analyses incorporating both extant and extinct genera of the Juglandaceae suggested that *Carya* originated in North America, and migrated to Eurasia during the early Tertiary via the North Atlantic land bridge. Fragmentation of the distribution caused by global cooling in the late Tertiary resulted in the current disjunction. The diversification rate of hickories in eastern North America appeared to be higher than that in eastern Asia, which is ascribed to greater ecological opportunities, key morphological innovations, and polyploidy.

## Introduction

As one of the typical phytogeographic disjunctions in the Northern Hemisphere, the eastern Asian (EA)-eastern North American (ENA) floristic disjunction pattern has received considerable attention (see 1-4 and references therein). This pattern is often explained by the boreotropical flora hypothesis [5]. A relatively continuous, homogenous mesophytic forest that spanned the Northern Hemisphere during the climatically warm mid-Tertiary became fragmented as global temperature cooled down in the late Tertiary and Quaternary [1,3,5-9]. Both the Bering land bridge (BLB) [10,11] and the North Atlantic land bridge (NALB) [12,13] probably contributed to the floristic intercontinental exchanges to form the boreotropical flora. Paleontological and molecular data suggest that BLB was used mostly by temperate taxa during the late Miocene and Pliocene (<10 Ma [4,8,14]). NALB has been viewed as a crucial route for the spread of subtropical and tropical taxa in the early Tertiary [5,6,13,15]. So far, most studies have focused on the temperate taxa in EA and ENA (see 1,3,14,16 and references therein); our understanding of the biogeographic relationships of the groups involving subtropical and tropical elements is still preliminary [3]. It is also noteworthy that species diversities within the two regions differ markedly. For taxa that distinctly distributed in EA and ENA, the former region usually has higher species richness than the latter [1,17,18]; this has been attributed to greater net speciation rate and accelerated molecular evolution in EA species [19]. However, several groups present a reverse biodiversity pattern, such as *Catalpa* (Bignoniaceae), *Lyonia* (Ericaceae), and *Carya* (Juglandaceae), in which ENA harbors more species [1]. Thus, to better understand the evolution of biodiversity in the two regions, an effort should be made to broaden the phylogenetic and biogeographic investigations on various disjunct taxa.

**Figure 1:**
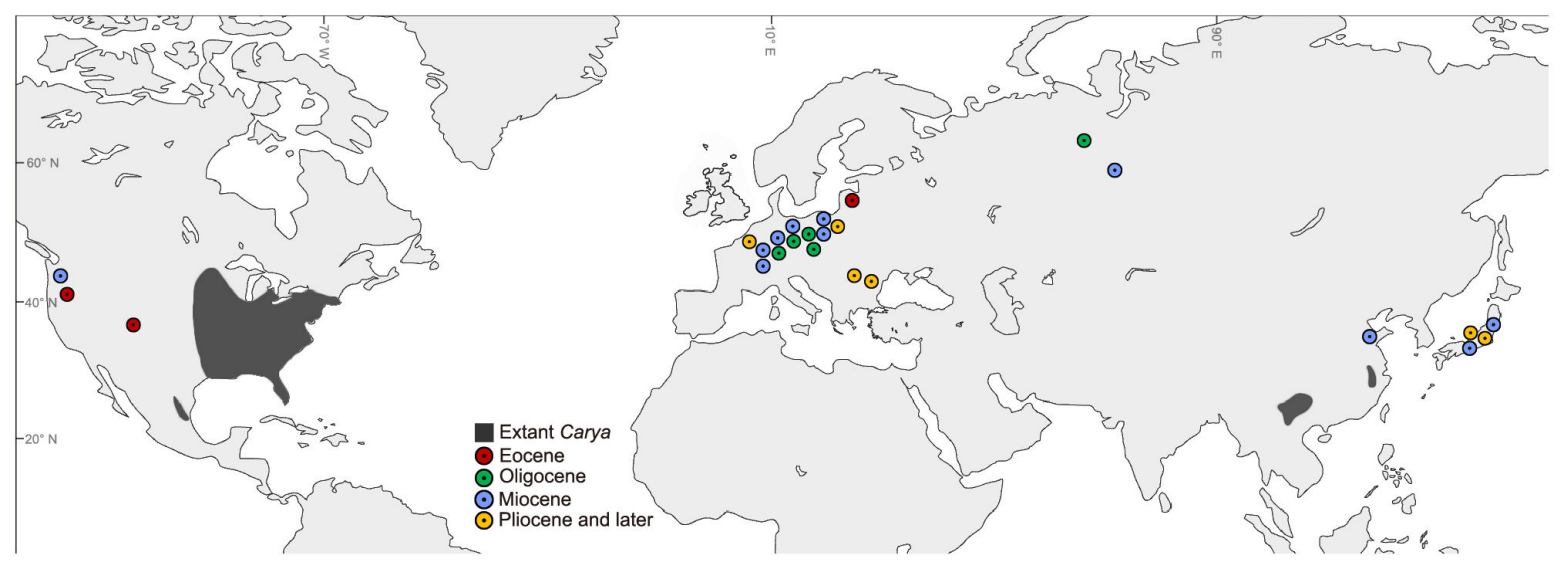
Geographic distribution of both extant and extinct *Carya* species. The black areas indicate the geographical distribution of extant *Carya* showing its disjunction between EA and ENA. Colored circles denote the locations of *Carya* fruit fossils (detailed information of each fossil in Table S4).

The hickory genus *Carya*, one of eight genera of the Juglandaceae, exhibits an intercontinentally disjunct distribution between EA and ENA (Figure 1). According to the records in *Flora of North America* [20] and *Flora of China* [21], *Carya* contains eleven species in ENA and four in EA. The ENA *Carya* *ovalis* (Wangenh.) Sarg. was treated as a synonym of *C* *. glabra* by *Flora of North America* [20], but this treatment is still controversial as both taxa quite distinct morphologically [22]. *Carya* *sinensis* Dode, endemic to southwestern China, was formerly treated as the monotypic genus *Annamocarya*, but it belongs to *Carya* according to molecular phylogenetic analyses [23,24]. *Carya* *poilanei* (Chev.) Leroy is also an accepted name of an EA hickory [25], which only has two specimen collections in Laos, collected in 1932 and 1937 [26]. Using ITS data, Xiang et al. [19] sampled three EA species and six ENA species and found that the EA clade was nested within the ENA grade; thus the two major clades of *Carya*, corresponding to geographical distribution, were not recovered. Based on morphology (64 characters) in combination with sequence data from three genomic regions (ITS, *trnL-F*, and *atpB-rbcL*), sampling the same nine species, two clades corresponding to the distributions of hickories in EA and ENA were recovered [23,24]. Because the importance of increased taxon sampling to enhance phylogenetic accuracy has been supported by several studies (see 27,28 and references therein), it is desired to test the reliability of phylogenetic relationships between EA and ENA species of *Carya* with an analysis including more species and more molecular loci.

According to the climate classification of Troll and Paffen (see 29), in EA, three hickory species are distributed in subtropical regions ( *C* *. cathayensis* , *C* *. hunanensis* , and *C* *. kweichowensis* ), and three in tropical regions ( *C* *. poilanei* , *C. sinensis*, and *C* *. tonkinensis* ). The ENA hickory species are distributed in the subtropical to temperate regions, with several species more prominent in temperate regions, such as *C* *. glabra* , *C* *. ovata* , and *C* *. illinoinensis* . Moreover, *Carya* also has a rich macrofossil record of fruits and leaves in North America [30,31] and Asia [30], as well as Europe [30,32,33]. Xiang et al. [34,35] have suggested that fossil taxa, especially those from sites outside the extant distribution, should be included in biogeographic analyses. Morphological data can be collected from both extinct and extant taxa, allowing the inclusion of fossils in the analyses and providing a more complete window to the evolutionary history that might not be revealed by molecular data alone [36,37]. The Juglandaceae contains at least eight extinct genera [30,38]. Some studies have attempted to reconstruct the genus-level phylogeny for Juglandaceae including extant and extinct genera [23,24,30]. It is feasible to explore the biogeography of *Carya* in the phylogenetic framework and geographic range of the whole family. Thus, *Carya* offers a remarkable opportunity to study biogeography and diversification of an EA-ENA disjunct group distributed across the temperate, subtropical and tropical regions in two continents.

The aims of our study were to (1) investigate phylogenetic relationships between EA and ENA species of *Carya* using eight plastid (*matK*, *rbcL-atpB*, *rpoC1*, *rps16*, *trnH-psbA*, and *trnL-F*) and nuclear (ITS and *phyA*) loci and an extensive taxon sampling, (2) reconstruct the historical biogeography of *Carya* by combining fossil, morphological, and molecular data, and (3) explore the causes of the unusual species richness pattern of EA and ENA *Carya*.

## Materials and Methods

### Ethics Statement

No special permits were required for this study because all samples were collected by researchers with introduction letters of IBCAS (Institute of Botany, Chinese Academy of Sciences) in Beijing, and this study did not involve endangered or protected species. Voucher specimens were deposited in the Herbarium, Institute of Botany, Beijing (PE).

### Sampling, DNA isolation, and sequencing

We sampled 17 accessions representing five species from EA, including *C. sinensis* (formerly treated as *Annamocarya* *sinensis* ), recorded in *Flora of China* [21], and eleven species from ENA as recognized in *Flora of North America* [20] as well as *C* *. ovalis* . Only *C* *. poilanei* was not sampled because no material was available for no more records have been found since 1937 [26]. Fifteen species representing all other seven genera of Juglandaceae were also sampled. All trees were rooted with *Rhoiptelea* *chiliantha* [23,24,39]. Voucher information and GenBank accession numbers are listed in Table S1.

Eight markers, including plastid (*matK*, *rbcL-atpB*, *rpoC1*, *rps16*, *trnH-psbA*, and *trnL-F*) and nuclear (ITS and *phyA*) loci were used to reconstruct the phylogeny of *Carya* and its relatives. Total DNA was extracted from silica-gel-dried leaf material using TianGen extraction kits (Beijing, China). The selected DNA regions were amplified with standard polymerase chain reaction (PCR). PCR primers used for amplifying and sequencing the eight plastid and nuclear loci are shown in Table S2.

### Molecular phylogenetic analyses

The resulting sequences were initially subjected to a search in BLAST (implemented by the National Center for Biotechnology Information (NCBI) website http://www.ncbi.nlm.nih.gov) against the GenBank nucleotide database to test for contamination and to confirm the targeted markers. All correct sequences were aligned using Clustal X version 2.0 [40], then adjusted manually in BioEdit version 4. 8. 10 [41]. Phylogenetic analyses were conducted using maximum likelihood (ML) and Bayesian inference (BI) methods. ML analyses were done at the Cyperinfrastructure for Phylogenetic Research (CIPRES; www.phylo.org) running RAxML-HPC2 on XSEDE [42,43] with the default settings except that the GTR + Г model was applied. Analyses of each independent data set produced no topological discordance with ML bootstrap proportions > 70%, and the eight data sets were therefore concatenated, yielding a combined molecular data set of 6024 characters (Dataset S1). Substitution model parameters were estimated separately for two partitions as plastid (*matK*, *rbcL-atpB*, *rpoC1*, *rps16*, *trnH-psbA*, and *trnL-F*) and nuclear (ITS and *phyA*) data partitions. The appropriate substitution models were determined using jModelTest version 1.0 [44]. Both partitions used the GTR + Г model. Bayesian inference was performed using MrBayes version 3.1.2 [45]. The MCMC algorithm was run for 5,000,000 generations with four incrementally heated chains, starting from random trees and sampling one out of every 1000 generations. Trees sampled before stable posterior probability (PP) values had been reached were excluded from consensus as a burn-in phase (initial 20% of the sampled trees), and the remaining trees were used to construct the Bayesian majority-rule consensus tree.

### Morphological phylogenetic analyses

A previously published morphological matrix [24] with 64 vegetative, floral, and fruit characters was modified to include more extant and extinct species, and the modified matrix (Table S3) was used to reconstruct a phylogenetic tree including both extant and fossil species of the Juglandaceae. Seven extinct genera have been included, with three fossils, representing extinct genera *Hooleya, Palaeocarya*, and *Paraengelhardia* newly added in this study. The coding of their morphological characters of the fossils was done according to the descriptions of Manchester [30]. Combined analysis of fossil and extant taxa with morphological and molecular data was performed using the DNA scaffold method [24] under Maximum Parsimony (MP) search on PAUP* version 4.0b10 [46], with the tree from our phylogenetic analysis of extant species used as the backbone constraint.

### Dating estimates

We estimated the divergence times within *Carya* using the combined molecular data set in BEAST version 1.7.4 [47]. BEAST employs a Bayesian MCMC to co-estimate topology, substitution rates, and node ages. Based on our phylogenetic analyses of extant and extinct genera in the Juglandaceae, four fossils were used for calibration purposes. Two fossils of extinct genera were used as minimum-age calibration points. *Paleooreomunneastoneana* is from the middle Eocene [48], and it is sister to the *Oreomunnea* - *Alfaroa* clade; we therefore used 40.4 to 48.6 Ma to calibrate the node age of the *Oreomunnea* - *Alfaroa* -*Engelhardia* clade [49]. *Polypteramanningii* is from the early Paleocene [50] and is sister to subtribe Juglandinae (including *Cyclocarya* , *Juglans* , and *Pterocarya* ) and *Platycarya* ; and we used its minimum age 64 Ma [49] to constrain the stem of the Juglandinae clade. Additionally, *Cyclocarya* *brownii* is an extinct species of *Cyclocarya* , which now comprises a single living species *Cyclocarya* *paliurus* in China [51]; thus we used 55.8 to 61.6 Ma [49] to constrain the stem age of *Cyclocarya* . These three calibration points were constrained with the lognormal prior distribution. In each implementation, the minimum age of each fossil was used as an offset value. In addition, we adjusted standard deviation and the mean in log space to match the maximum bound of each fossil with the 97.5% of the distribution [52]. An age of 84 Ma was used to constrain the maximum root age of the phylogeny, which is the age of the earliest Fagales fossil *Antiquacupula* [53-55], with a normal prior distribution (standard deviation, 0.5) [49,55].

The data set was partitioned based on plastid or nuclear genomes. According to the results of jModelTest version 1.0, we used the GTR + Г model as described above. A starting tree with branch lengths satisfying all fossil prior constraints was created using the program r8s version 1.7 [56] by non-parametric rate smoothing (NPRS) [57]. Beast analysis was performed with an uncorrelated lognormal relaxed molecular clock model for 100,000,000 generations. Adequate sampling and convergence of the chain to stationarity were confirmed by inspection of MCMC samples using Tracer ver. 1.5 [58], as all parameters of the effective sample size (ESS) values were greater than 200, suggesting sufficient sampling in our BEAST analysis. A maximum-credibility tree, representing the maximum a posteriori topology, was calculated after removing 50% burn-in with TreeAnnotator version 1.5.4 [59], which also calculated the mean ages and 95% highest posterior density (95% HPD) intervals for each node.

### Biogeographic analyses

After estimating the phylogenetic relationships including fossils, we derived a summary tree at the generic level. Subsequently, according to the records of fruit fossils of *Carya* (Table S4) [30], we mapped three branches to represent the distributions of *Carya* in the Eocene, Oligocene, and early Miocene, respectively. Each branch was inserted manually in the newick chronogram along the stem lineage of *Carya*, with placement according to their position in the geologic time scale. Using this phylogeny, we employed the dispersal-vicariance analysis (DIVA) [60] and a maximum likelihood based method LAGRANGE [61,62] to construct the biogeographic history of *Carya*. Biogeographic analyses were also conducted without fossil information. Based on the geographical distribution patterns of extant and extinct genera of Juglandaceae (Table S5), we categorized the distributions into the following endemic areas: North America (A), Europe (B), and Asia (C).

### Diversification rate analyses

To visualize the temporal diversification in *Carya*, we produced lineage-through-time (LTT) plots using the R package APE [63]. Plots were produced for 1000 sampled trees from the converged BEAST trees for entire *Carya* genus, then its EA lineages, and its ENA lineages, respectively. The net diversification rate (r) for each clade within *Carya* was also estimated following equation (7) of Magallón and Sanderson [64] for crown groups under the assumptions of either no extinction (ε = 0) or high relative extinction (ε = 0.9) to evaluate relative species diversification between clades. Calculations were done using the R package LASER [65] and the crown age of each clade was inferred from the BEAST analysis (Table 1).

**Table 1:** Net diversification rates (*r*) estimated for *Carya* based on the equation (7) of Magallón and Sanderson [59].

Clade	Crown group age (95% HPD) Ma	Number of species	*r* (ε = 0)	*r* (ε = 0.9)
				
*Carya*	21.58 (11.07-35.51)	17	0.0991	0.0418
ENA *Carya*	10.10 (5.26-16.78)	12	0.1774	0.0682
EA *Carya*	12.81 (5.60-22.04)	5	0.0716	0.0218

Note: ε, the relative extinction rate

## Results

### Phylogenetic analyses

**Figure 2:**
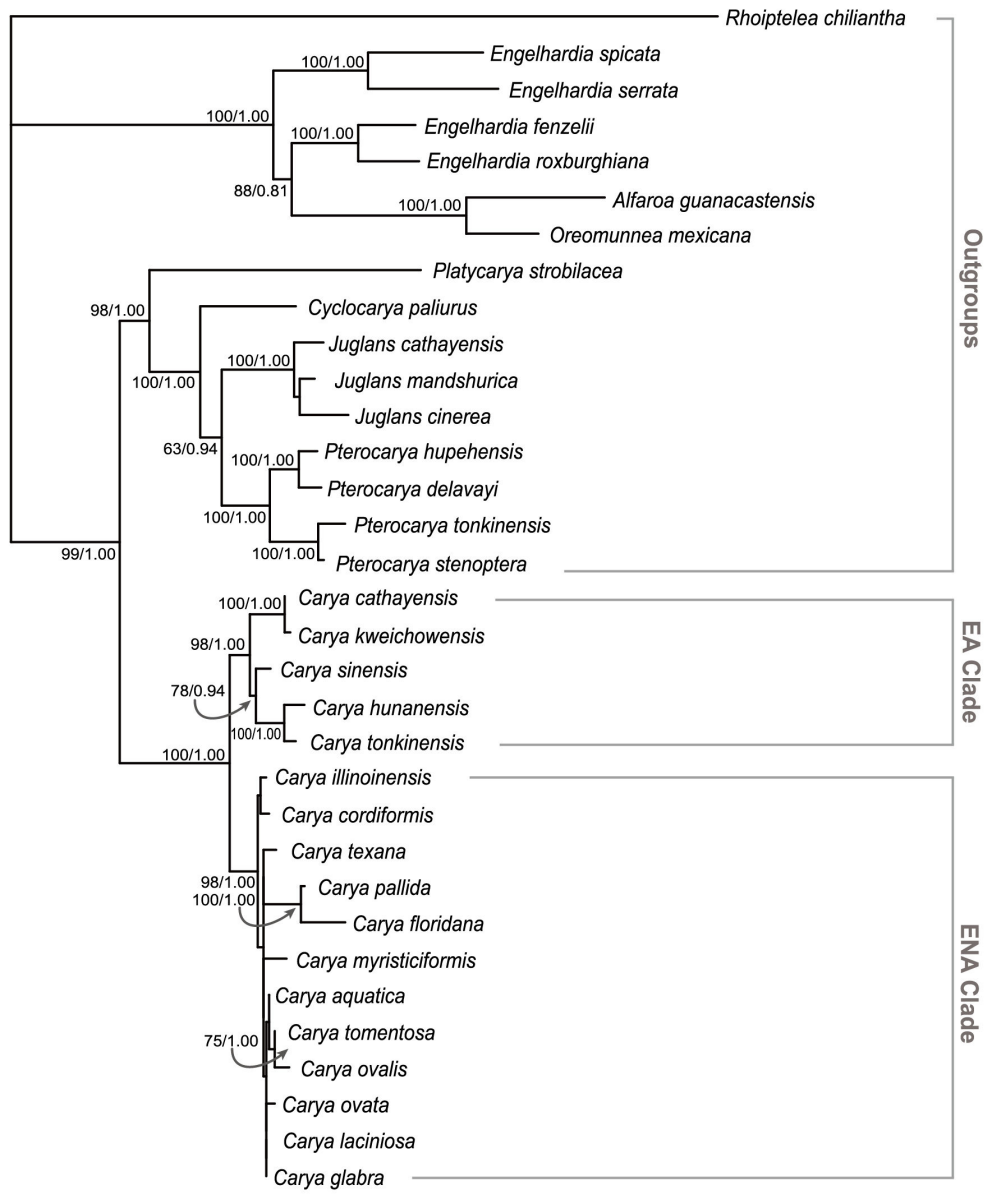
Phylogeny of *Carya* based on the combined molecular data set. ML bootstrap support > 75% and Bayesian posterior probability > 0.80 are indicated at the nodes, respectively. The topologies of strict consensus trees from maximum likelihood and Bayesian inference are same.

A total of 200 sequences were newly obtained (Table S1). ML and BI result in almost the same trees (Figure 2). Within the Juglandaceae, two major clades are identified. One includes *Alfaroa* , *Engelhardia*, and *Oreomunnea* (maximum likelihood bootstrap support, MLBS 100%; PP 1.00). *Carya*, *Cyclocarya* , *Juglans* , *Platycarya* , and *Pterocarya* form the other clade (MLBS 99%; PP 1.00), in which *Carya* is sister to the clade of the remaining genera*.*

*Carya* is strongly supported as monophyletic (MLBS 100%; PP 1.00), and two major clades are well supported, which correspond to an EA group (MLBS 98%; PP 1.00) and an ENA group (MLBS 98%; PP 1.00), respectively. Within the EA group, *C* *. cathayensis* and *C* *. kweichowensis* are in a clade that is sister to a subclade of the remaining three species. In the ENA group, the relationships among ENA species are poorly resolved; nevertheless *C* *. ovalis* and *C* *. tomentosa* form a clade with moderate or strong support (MLBS 75%; PP 1.00).

**Figure 3:**
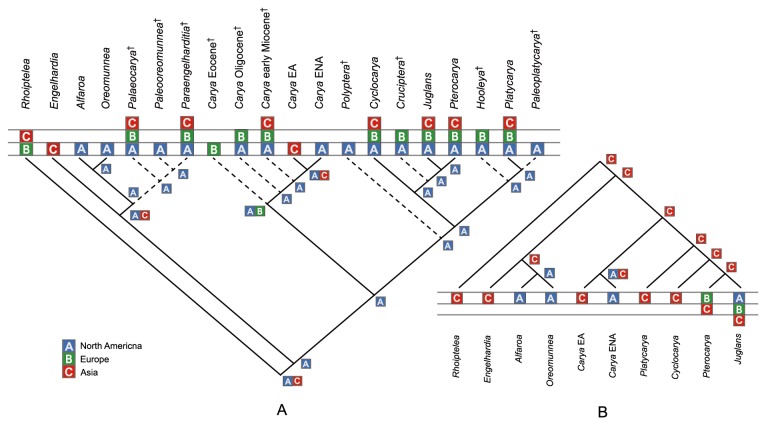
Ancestral area reconstructions for *Carya* using DIVA. A: Inference with fossil taxa; B: Inference without fossil taxa. Fossil taxa are indicated by cross symbols after their names. The areas of endemism are defined for both analyses: A, North America; B, Europe; C, Asia.

**Figure 4:**
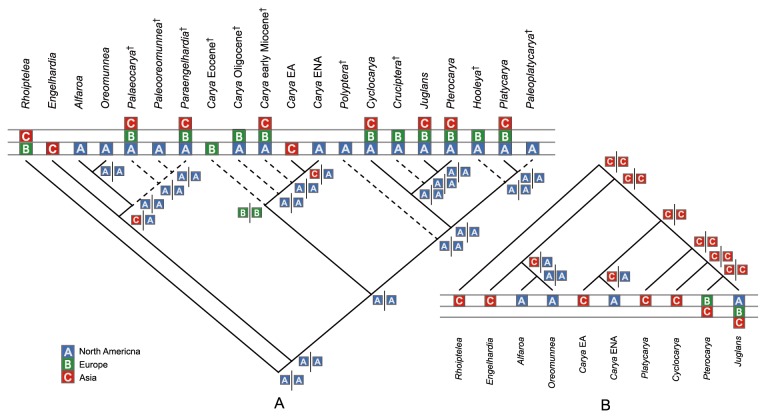
Ancestral area reconstructions for *Carya* using LAGRANGE. A: Inference with fossil taxa; B: Inference without fossil taxa. Fossil taxa are indicated by cross symbols after their names. A slash in the results of LAGRANGE indicates the split of areas in two daughter lineages. The areas of endemism are defined for both analyses: A, North America; B, Europe; C, Asia.

The placements of extinct genera are resolved in our analysis (Figures 3A and 4A). Three extinct genera, *Paleooreomunnea*, *Palaeocarya*, and *Paraengelhardia* form a clade, sister to the extant *Alfaroa* - *Oreomunnea* clade. The extinct *Paleoplatycarya* and the extant *Platycarya* form a clade, which is sister to the extinct *Hooleya*. The extinct *Cruciptera* is sister to the clade containing extant *Juglans* and *Pterocarya* . The extinct *Polyptera* and the extant *Carya* are in a clade.

### Historical biogeography and divergence times

**Figure 5:**
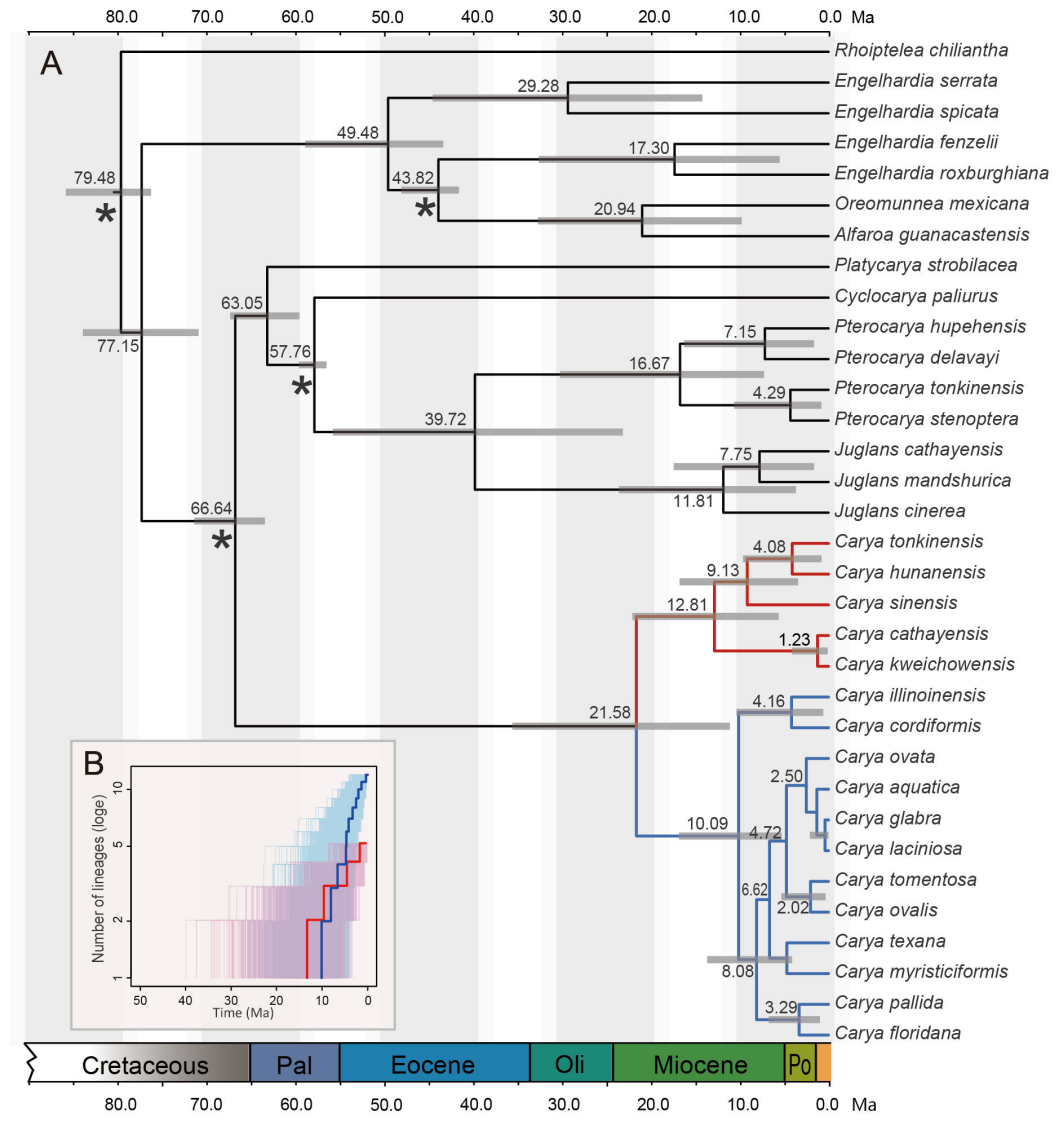
BEAST chronogram and LTT plots of *Carya*. A: The chronogram of C*arya* inferred from the combined molecular data set. Gray bars represent the 95% HPD intervals of node ages. Calibration points are indicated with asterisks. B: Lineage-through-time (LTT) plots for *Carya*. The thin lines indicate the 1000 random trees from the best-fitting model obtained in BEAST (EA clade: pink; ENA clade: light blue); the bold lines correspond to the maximum credibility tree from the BEAST analysis (EA clade: red; ENA clade: blue).

The chronogram of *Carya* is shown in Figure 5. Based on our dating estimates, the age of the Juglandaceae is 77.15 (95% HPD 70.76-83.76) Ma, and the time of origin of *Carya* is 66.64 (95% HPD 63.30-71.26) Ma. The split between the EA and ENA clades occurred at 21.58 (95% HPD 11.07-35.51) Ma. EA and ENA *Carya* began to diversify at 12.81 (95% HPD 5.60-22.04) Ma and 10.10 (95% HPD 5.26-16.78) Ma, respectively.

Based on the phylogenetic tree including both extant and extinct genera of the Juglandaceae, the DIVA and LAGRANGE analyses suggest that *Carya* originated in North America (Figures 3A and 4A). In contrast, results from the analyses for the only extant genera of Juglandaceae, however, indicate that the ancestral area of *Carya* is Asia (Figures 3B and 4B).

### Diversification rates

The lineage-through-time (LTT) plots for the EA and ENA clades are shown in Figure 5. Compared with the EA clade, the ENA clade had a dramatic accumulation of lineages after the middle Miocene. Our diversification rate estimates based on net diversification rates (r) following equation (7) of Magallón and Sanderson [64] also showed that the ENA clade had a significantly higher net diversification rate (r) than the EA clade after the middle Miocene (ε = 0, 0.0863 > 0.0389; ε = 0.9, 0.0682 > 0.0218; Table 1).

## Discussion

### Phylogeny

Our phylogenetic analyses indicate that the Juglandaceae comprise two major clades. *Carya* is sister to the *Cyclocarya* - *Juglans* - *Platycarya* - *Pterocarya* clade. The results are congruent with those of previous studies [23,24]. Within *Carya*, two monophyletic groups corresponding to geographic distributions (EA and ENA) are well supported (EA: MLBS 98%; PP 1.00; ENA: MLBS 98%; PP 1.00). Given that incomplete taxon sampling can sometimes influence phylogenetic inference [66], our analyses with an extensive sampling further clarified the correlation of phylogenetic relationships and geographic distributions in *Carya*. Although Stone [20] treated *C* *. ovalis* as a synonym of *C* *. glabra* , our analyses indicate that *C* *. ovalis* is sister to *C* *. tomentosa* . Morphologically, *C* *. ovalis* differs from *C* *. glabra* in many characters, such as reddish petioles (*vs.* plain), fruit stipe rarely present (*vs.* often present), dehiscence of the fruits to the base (*vs.* only at apex or to middle), mature husk warty and dull (vs. smooth and shining), and nut shell ridged and thin (*vs.* not ridged and thick) [22].

Seven extinct genera were placed into the phylogenetic framework of extant species using the DNA scaffold method. Four of these, i.e., *Cruciptera*, *Paleoplatycarya*, *Paleooreomunnea*, and *Polyptera*, were also used by Manos et al. [24]. The placement of *Cruciptera* was congruent with that in previous studies [23,24,30]. *Paleooreomunnea* and the two newly added extinct genera *Palaeocarya* and *Paraengelhardia* formed a clade showing a close relationship with an extant clade that includes *Alfaroa* , *Engelhardia*, and *Oreomunnea* . The sister relationship between *Paleoplatycarya* and *Platycarya* from our inferences was also recognized in the study of Manos et al. [24]. For the extinct genus *Polyptera*, previous studies suggested it sister to the clade containing Juglandinae ( *Cyclocarya* , *Juglans* , and *Pterocarya* ) and *Carya* [50,67]. Our results support *Polyptera* with a closer relationship to Juglandinae than *Carya*. This may be ascribed to the similarity of some morphological characters. For example, both *Polyptera* and *Cyclocarya* have the pollen with equatorial pores readily distinguished from the subequatorial pores of *Carya* [23]; the production of a large number of fruits on a spicate infructescence in *Polyptera* is similar to the situation in *Cyclocarya* and *Pterocarya* ; and both *Polyptera* and *Cyclocarya* have a disk-like wing oriented perpendicular to the nut axis [50].

### Evolution of intercontinental disjunctions in *Carya*

Both DIVA and LAGRANGE analyses including fossil information suggested North America as the ancestral area for *Carya*, but analyses without fossil information indicated that *Carya* originated in Asia. Because *Carya* has a pollen type known as *Caryapollenites*, which was suggested to be derived directly from *Normapolles* [30,68], the origin of *Carya* should be within or at least near the distribution of the *Normapolles* complex, which defines a middle to late Cretaceous province including ENA and Europe. Therefore, the palynological evidence supports the inference by the analyses with fossil information for an origin in North America. Although *Carya* is not native in Europe today, according to the fossil evidence, Europe was a center of diversity of *Carya* during the Tertiary, especially during the Miocene, with some species extending even to the Quaternary [30]. The difference in ancestral area reconstruction is likely due to the much broader geographic distributions of the fossils. Hence, the fossil information is particularly important for reliably reconstructing the historical biogeography of *Carya* and the following discussion is based on the result inferred by the analyses that included occurrence data from the fossil records.

Our result for the age of the Juglandaceae, as 77.15 (95% HPD 70.76-83.76) Ma, is similar with the outcome of Sauquet et al. [49] inferred with the default calibrations, as 72.6 (95% HPD 64.4-81.3) Ma. Our estimations are also congruent with those of Manos et al. [24] who constrained the minimum age of the Juglandinae clade at 58 Ma. The age of the subfamily Juglandoideae (including *Platycarya* , *Carya*, *Cyclocarya* , *Juglans* , and *Pterocarya* ) is herein estimated as 66.64 [95% HPD 63.30-71.26] Ma, which is almost the same as the estimation of 65.0 [95% HPD 58.3-73.0] Ma by Sauquet et al. [49] using the default calibrations. In addition, based on the presence of fossil pollen which is similar to that of extant *Carya* of western Europe and North America in the Late Paleocene. Manchester [30] noted that the first hint of the Carinae (the subtribe including *Carya*) occurred in the Paleocene. This also supports our estimate for the stem age of *Carya* dating back to the Paleocene (66.64 [95% HPD 63.30-71.26]). Therefore, the divergence date estimates inferred by our calibration points should be reliable to reconstruct the biogeographic history of *Carya*.

**Figure 6:**
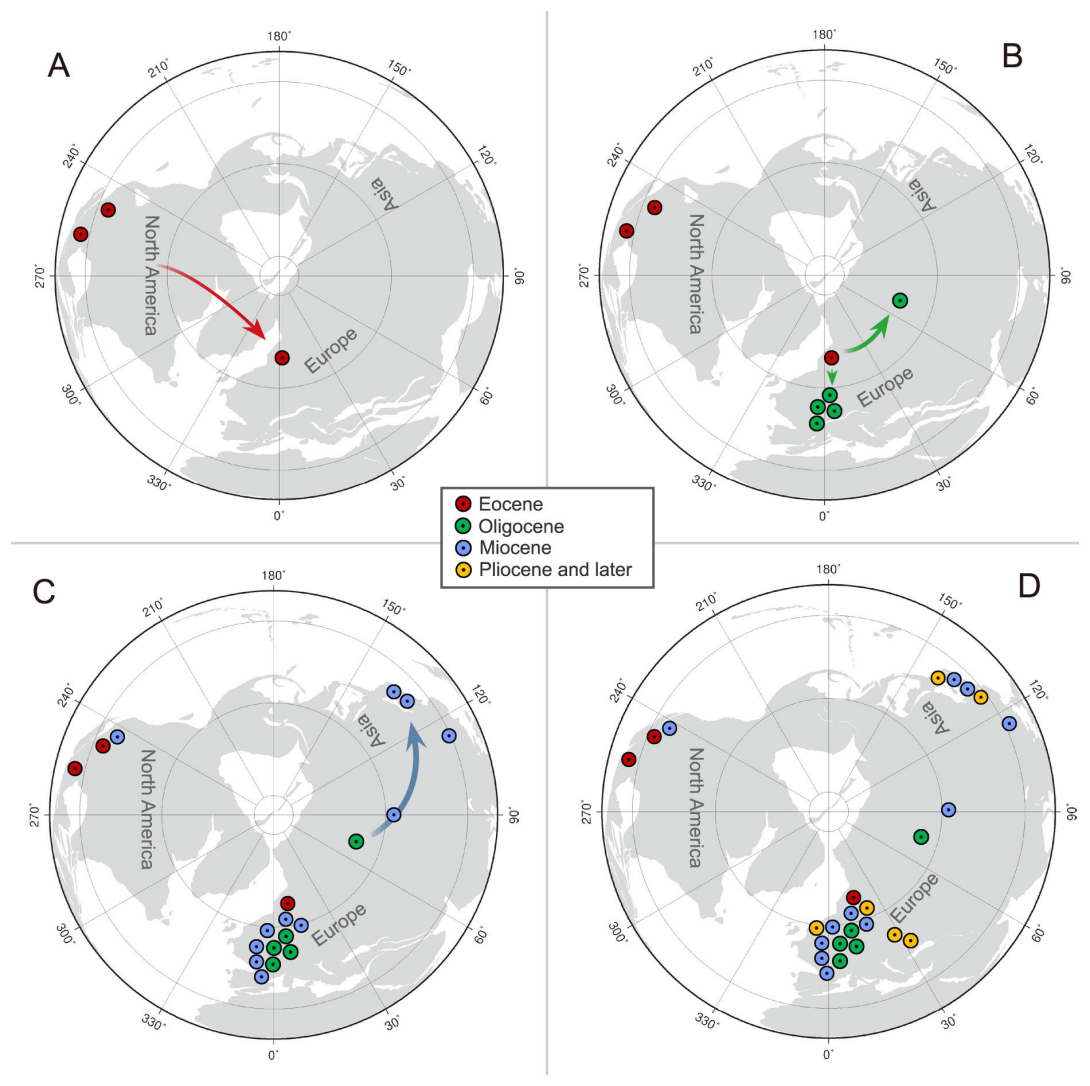
Hypothetical migratory routes of *Carya*. A: Eocene; B: Oligocene; C: Miocene; D: Pliocene and later. The colored arrows and circles indicate the migratory directions and *Carya* fruit fossil records, respectively.

The age estimates and biogeographic analyses suggest *Carya* originated in North America during the early Paleocene. Previous studies have inferred that a relatively uniform, warm climate was prevalent in the Northern Hemisphere during the early Tertiary, which allowed populations of the boreotropical flora to move between continental areas via northern land bridges [4-6,15,34]. Both the BLB and NALB played important roles in plant exchanges during the Tertiary, but with differential importance in different geologic times [34]. Previous studies suggest that the floristic migration via the NALB was possible during the Paleocene and Eocene, and it was more likely to be used by thermophilic species [5,6,13,15]. The BLB was used mostly by temperate taxa during the late Miocene and Pliocene (<10 Ma [4,8,14]). The divergence time of the two *Carya* clades indicates that *Carya* likely migrated from North America to Europe via the NALB like other thermophilic groups, such as *Ampelopsis* (Vitaceae) [69], *Cercis* (Fabaceae) [70], *Cornus* (Cornaceae) [35], Malpighiaceae [71], and *Quercus* (Fagaceae) [72]. The distribution pattern of *Carya* fossils also supports this inference. Given that the NALB connected North America and western Europe, *Carya*-like pollen was abundant in both continents and nuts have been confirmed from the late Eocene of North America [31]. Oligocene and younger macrofossils of *Carya* have been confirmed in western Europe, supporting an Oligocene regional diversification. In contrast, the earliest eastern Asian *Carya* fruit fossils occurred only in the Miocene (Figure 6). The group might have entered Asia from Europe as the Turgai seaway receded, and/or from North America via the BLB.

Starting in the Miocene, there was a distinct climatic cooling period across the high latitude areas of the Northern Hemisphere, which may have resulted in a reduction of the distribution of forests [5]. According to the maps of the past geographic ranges of *Carya* presented by Manchester [30] and Mai [32], *Carya* was more broadly distributed in the Miocene, with occurrences including Europe, Siberia, and western North America, where the genus is absent today. The present disjunct distribution of *Carya* between EA and ENA can be explained by extinctions in large parts of its former ranges. Therefore, extinctions caused the range fragmentation of *Carya*, ultimately leading to the modern intercontinental distribution between EA and ENA. Extinction events could have extirpated the old stem relatives that diverged prior to the extant crown radiation, leaving a phylogeny that includes only extant taxa with long stems and species-rich crowns [73]. A remarkably long “temporal gap” between the *Carya* stem age (the Early Cretaceous) and the beginning of the extant radiation (the Early Miocene) is detected, which supports that *Carya* has been experienced several extinction events during the Tertiary.

### Higher diversification rate in the ENA Clade

Our results of diversification analyses showed that ENA *Carya* has a significantly higher diversification rate than EA since the Miocene. Both environmental [74-77] and biological attributes [78-80] may directly facilitate species diversification [81-83]. The formation of geologic barriers is one of the most important factors driving speciation [84-87]. For ENA, after being largely eroded to plains by the end of the Mesozoic, renewed uplift of Appalachian Mountains occurred during the Tertiary [88,89]. According to the data from the sedimentation rates and fault ages, this major uplift occurred during the late Oligocene to Miocene, followed by a quiescent interval, in which erosion occurred that lasted until the end of Miocene [90-96]. These dramatic changes of environments offered many ecological opportunities, contributing to divergence in several taxa in this area [87,97-103]. This is consistent with our inference that ENA *Carya* underwent a high diversification rate since the late Miocene. On the other hand, EA *Carya* taxa are primarily distributed in central, south-central, and south-eastern China, a region that was relatively stable tectonically since the late Tertiary [104,105]. This stability allowed these areas to be refugia, facilitating the survival of many relict plant lineages during the cold period of the Tertiary. Once climatic conditions improved, not all refugial species were able to migrate out but a significant proportion of species remained restricted [105-108]. Hence, these species exhibit disjunct distributions and relatively lower diversification rates [109]. The EA *Carya* lineage is a typical example of this pattern. Although during [110-112] or immediately before [113,114] the Pliocene and Pleistocene, the major uplift of the Tibetan Plateau took place in EA, which created a vast array of new habitats across wide elevational ranges in the eastern fringe of the Tibetan Plateau [115], the distribution areas of EA *Carya* are not part of this region.

ENA *Carya* species have terminal bud scales, while the EA species have naked terminal buds (excluding *C. sinensis* with pseudo-valvate terminal bud scales). The presence of bud scales is considered to be an adaptation for cooling climates [116], so the ENA species may be better adapted for the cooler temperate climate since the Miocene. At least six ENA *Carya* species ( *C* *. floridana* *,* *C* *. glabra* *,* *C. myristiciformis* *,* *C* *. pallida* *,* *C* *. texana* , and *C* *. tomentosa* ) are tetraploid (*n* = 32) [20,117,118], and the only confirmed count for EA *Carya* species is diploid ( *C* *. cathayensis* , *n* = 16 [20,119]). Polyploidization may allow species to adapt to dramatic changes in the environment [118,120,121], so that polyploid lineages may have higher diversification rates than that of diploid lineages [122,123]. Furthermore, sympatric *Carya* species in southeastern Ohio have shown distribution replacement especially concerning topography and its associated microhabitats [124]. Thus, the richness of ecological opportunities, key morphological innovations, and polyploidy may have been responsible for the high diversification rate of ENA *Carya*.

## Supporting Information

Table S1 Taxa, voucher identification, and GenBank accession numbers for samples included in this study. (XLS)

Table S2 PCR and sequencing primers used in this study. (XLS)

Table S3 Morphological characters matrix including both extant and extinct species of the Juglandaceae. (XLS)

Table S4 Detailed information of extinct *Carya* species with fruit fossils. (XLS)

Table S5 Extinct taxa with fruit fossils in other genera of the Juglandaceae. (XLS)

Dataset S1 Data matrix. The aligned sequence data is presented in Nexus format.
(TXT)
